# PReS-FINAL-2247: Confidence of UK general peadiatric trainees in musculoskeletal clinical assessment and preferences for future teaching resources

**DOI:** 10.1186/1546-0096-11-S2-P237

**Published:** 2013-12-05

**Authors:** E Smith, M Cruikshank, H Dean, HE Foster, S Jandial

**Affiliations:** 1 Paediatric Rheumatology, Great North Children's Hospital, Newcastle Upon Tyne, UK; 2 Institute of Cellular Medicine, Newcastle University, Newcastle Upon Tyne, UK

## Introduction

Musculoskeletal (MSK) problems in children and adolescents are common (1-4) and may represent serious life threatening disease (5-7). Many doctors have low confidence in examining children's joints, stemming from MSK teaching not being core in many training programmes (8). In an attempt to address this, paediatric MSK competencies were introduced into the Royal College of Paediatrics and Child Health (RCPCH) training curriculum in 2007 and assessment of MSK knowledge and clinical skills was included in the mandatory professional clinical examinations for all paediatricians in 2009 (i.e. Membership of the RCPCH (MRCPCH) clinical examination).

## Objectives

To examine:

[1] Self-rated confidence in paediatric MSK clinical assessment in general paediatric trainees in relation to their ability to undertake MSK station of the MRCPCH clinical examination.

[2] Access to MSK teaching. 

[3] Which types of educational resources trainees use when preparing for the examination. 

[4] The preferred format for future paediatric MSK teaching resources.

## Methods

An anonymous Survey Monkey e-mail questionnaire was disseminated to UK paediatric trainees, from the North of England and South East of Scotland.

## Results

35 trainees completed the survey. Going into the examination, trainee confidence in undertaking the MSK station was lower than for the cardiovascular, respiratory, abdominal and developmental stations but marginally better than the neurological station. 20% of trainees found it 'hard' to get access to face-to-face teaching before the exam, and a further 66% felt that it 'took some effort'. When preparing for the MSK station, trainees reported using the following teaching resources (in decreasing order of frequency); bedside teaching, pGALS DVD, textbooks, MRCPCH clinical examination revision course, attending paediatric rheumatology clinics or a joint injection list. 46% and 34% of trainees felt that a 1-day MSK revision course or e-learning module would respectively best prepare them for the MSK station of the examination.

## Conclusion

From these responses it is clear that self-rated paediatric trainee confidence in undertaking the MSK station of the MRCPCH clinical examination remains low as compared to other bodily systems. Although the MRCPCH provides impetus for trainees to engage in learning about MSK examination, trainees reported a variation in access to teaching prior to the examination. We know that access to traditional face-to-face MSK clinical teaching is limited; many trainees do not have the opportunity to rotate through rheumatology and there are too few paediatric rheumatologists to provide teaching sessions for trainees. Trainees currently use of a wide range of MSK teaching methods but preference for future teaching resource development is a 1-day paediatric MSK revision course or e-learning module. The results of this survey highlight the need for improved access to MSK teaching, and their stated preferred method of educational delivery, which if introduced, we anticipate will facilitate more confident, and prompt recognition of MSK disease by the next generation of paediatricians.

## Disclosure of interest

None declared.

